# Generation of Glucose-Responsive Functional Islets with a Three-Dimensional Structure from Mouse Fetal Pancreatic Cells and iPS Cells *In Vitro*

**DOI:** 10.1371/journal.pone.0028209

**Published:** 2011-12-01

**Authors:** Hiroki Saito, Masaki Takeuchi, Kazuhiro Chida, Atsushi Miyajima

**Affiliations:** 1 Institute of Molecular and Cellular Biosciences, University of Tokyo, Tokyo, Japan; 2 Graduate School of Agricultural and Life Sciences, University of Tokyo, Tokyo, Japan; University of Bremen, Germany

## Abstract

Islets of Langerhans are a pancreatic endocrine compartment consisting of insulin-producing β cells together with several other hormone-producing cells. While some insulin-producing cells or immature pancreatic cells have been generated *in vitro* from ES and iPS cells, islets with proper functions and a three-dimensional (3D) structure have never been successfully produced. To test whether islets can be formed *in vitro*, we first examined the potential of mouse fetal pancreatic cells. We found that E16.5 pancreatic cells, just before forming islets, were able to develop cell aggregates consisting of β cells surrounded by glucagon-producing α cells, a structure similar to murine adult islets. Moreover, the transplantation of these cells improved blood glucose levels in hyperglycemic mice. These results indicate that functional islets are formed *in vitro* from fetal pancreatic cells at a specific developmental stage. By adopting these culture conditions to the differentiation of mouse iPS cells, we developed a two-step system to generate islets, i.e. immature pancreatic cells were first produced from iPS cells, and then transferred to culture conditions that allowed the formation of islets from fetal pancreatic cells. The islets exhibited distinct 3D structural features similar to adult pancreatic islets and secreted insulin in response to glucose concentrations. Transplantation of the islets improved blood glucose levels in hyperglycemic mice. In conclusion, the two-step culture system allows the generation of functional islets with a 3D structure from iPS cells.

## Introduction

The pancreas consists of exocrine and endocrine compartments. The endocrine compartment, known as islets of Langerhans, is composed of several types of endocrine cells including, α, β, δ, and PP cells, which secrete glucagon, insulin, somatostatin, and pancreatic polypeptide, respectively. The islet exhibits a unique three-dimensional (3D) structure that differs between species. Rodent islets are characterized by a spherical core of β cells surrounded by a thin layer of non-β cells, which called mantle-core, whereas α and β cells are in close contact throughout human islets. During embryonic development, the expression of pancreatic proteins such as glucagon and amylase starts at around mouse embryonic day (E) 13.5, followed by the expression of other pancreatic proteins including insulin. Subsequently, distinct 3D structures of islets and exocrine acini are formed, though it remains unclear how such structures are formed. Neurogenin 3 (Ngn3) is an essential transcription factor for pancreatic development and Ngn3-positive cells are considered as islet progenitors [1]. Those cells are present in fetal pancreas but disappear postnatally in normal development [2]. In experimental pancreatic injuries, such as partial pancreatectomy or ablation of β cells in adults, differentiated β cells proliferate to repair the damage [3]. Moreover, the number of pancreatic progenitors in the fetus determines the size of the adult pancreas [4]. These results together suggest that islet-genesis occurs only in the fetus.

Pancreatic islets play a central role in glycemic control. Type I diabetes is a disease that destroys β cells resulting in insulin deficiency, and patients with type I diabetes depend on the administration of insulin for survival. However, fluctuations of blood glucose levels can lead to complications and the transplantation of islets is a promising therapeutic option for such patients. With this protocol, islets isolated from donors are infused via the portal vein. As the transplanted islets secrete insulin in response to blood glucose levels, islets are a functional unit that can ectopically regulate the blood glucose level [5]. However, donor shortage is a major problem and a system of preparing a sufficient quantity of functional islets is required. To this end, efforts have been made to generate islets or β cells from various cell sources such as pancreatic ducts and embryonic stem cells (ES) [6]–[12]. The advent of induced pluripotent stem cell (iPS) technology has further fueled these efforts. Although there are some reports of the development of insulin-producing cells *in vitro*, no functional islets have ever been produced *in vitro*.

In organ culture of human fetal pancreas at 6–9 weeks of age, islets were shown to develop *ex vivo* [13], [14], suggesting that fetal pancreatic cells have the potential to form islets *in vitro*. In this paper we show that islets are formed between E16.5 and E18.5 in mice, and that cultivation of the pancreatic cells at E16.5 allows the formation of functional islets with a structure similar to adult islets. By adopting this culture system to immature pancreatic cells derived from mouse iPS cells, we show that functional islets can be generated *in vitro*.

## Materials and Methods

### Mice

C57BL/6 and Akita mice were from Japan SLC, Inc. and bred under specific pathogen-free conditions. The mice were maintained and mated in the institutional animal facility according to the guidelines of the University of Tokyo. The experimental procedures in this study were approved by the Committee for Animal Experiments in the Institute of Molecular and Cellular Biosciences University of Tokyo (approval number is 23003).

### Antibodies

Primary antibodies used and dilutions were; guinea pig anti-insulin (Dako, 1/200), rabbit anti-glucagon (Dako, 1/200), rabbit anti-somatostatin (Dako, 1/200), rabbit anti-pancreatic polypeptide (Dako, 1/200), goat anti-C-peptide (Linco, 1/200), rabbit anti-amylase (Sigma, 1/200), rabbit anti-CK19 [15], [16], guinea pig IgG isotype control (INTER-CELL TECHNOLOGIES INC, 1/200), and rabbit IgG isotype control (Vector, 1/200). The secondary antibody used was biotinylated anti-guinea pig IgG (Vector, 1/200). Thirdly antibodies used were; Alexaflour 488-conjugated anti-rabbit IgG (Molecular Probes, 1/200), Alexaflour 555 anti-goat IgG (Molecular Probes, 1/200), and SA-conjugated Alexafloura 594 (Molecular Probes, 1/200).

### Preparation of Pancreatic Cells

Single-cell suspensions were prepared from the pancreata of E14.5, E16.5, E18.5, neonate, P7, and adult mice. The pancreata of the neonate, P7 and adult mice were carefully dissected under a microscope and minced with scissors. Pancreatic tissues were placed in Ca^2+^- Mg^2+^- free PBS containing 2 mg/ml collagenase type V (Sigma) and mixed by gentle pipetting every 3 minutes during incubation for 10 min at 37°C. Digested pancreatic cells were filtrated and washed in medium containing 10% FCS before the cultivation. Cell viability was greater than 90% (fetal, neonate) or 70% (P7, adult) as assessed by trypan blue dye exclusion.

### Pancreatic Cell Culture

Digested pancreatic cells were seeded on a gelatin-coated 6-well plate at 5×10^4^ cells/cm^2^. Our standard culture medium is a 1∶1 mixture of Dulbecco's modified Eagle's medium and F-12 (Gibco) with 5% fetal bovine serum (Gibco), Nicotinamide (10 µmol/l, Sigma), β-mercaptoethenol (50 µmol/l, Sigma), HEPES (5 mmol/l, Gibco), and Gentamycin (50 µg/ml, Sigma). At 24 h after seeding, the medium was changed to the standard medium supplemented with epidermal growth factor (EGF) (20 ng/ml, Peprotech), glucagon-like peptide (GLP)-1 (10 ng/ml, Sigma), and γ-insulin (10 µM, Wako). At day 12 of culture, cultured cells were used for RT-PCR analysis, histological analysis, and transplantation. For the histological analysis of frozen sections and transplantation, cultured cells were detached from the culture dish by pipetting with 2 mg/ml collagenase type V/PBS, taking care not to disrupt the 3D structure of islets and ducts formed *in vitro*.

### iPS Cell Culture

iPS cells were maintained in a standard ES/iPS cell maintenance medium, DMEM/F12 with 15% KSR and 200 U ESGRO (mLIF), according to the protocol previously described [17]. iPS cells were dissociated into single cells using TripLE (Invitrogen) and plated on a gelatin-coated dish for 30 min to eliminate feeder cells. After the plating was repeated, non-adherent cells were harvested and seeded on a collagen type IV-coated dish (IWAKI) to induce their differentiation into pancreatic cells. The differentiation of iPS cells into pancreatic cells was performed as described previously [18]–[20] with some arranges and modifications to seeding density and duration of culture. Briefly, activin, retinoic acid, bFGF, bFGF+Nicotinamide were added sequentially to iPS cells (see **Fig. S4**). The pancreatic cells differentiated from iPS cells were dispersed into a single cell suspension using 2 mg/ml collagenase/PBS and then seeded onto a gelatin-coated dish to induce the formation of islets with the protocol used for fetal pancreatic cells described above.

### Histology

Tissues, cultured cells, and transplanted kidneys were fixed in 4% (wt/vol) paraformaldehyde, and stained with antibodies as described in the Antibodies section. Tissues were embedded in OCT compound (Tissue-Tek), and frozen sections (8 µm) were prepared. For frozen sections of cultured cells, the cells were detached from a culture dish as a sheet using collagenase. The detached cells were fixed and embedded in OCT compound.

### Cell transplantation and glucose level

The blood glucose levels were measured under nonfasting conditions and were about 250 mg/dL in normal mice by the assay system we used. Hyperglycemia was induced in C57BL/6 mice by intraperitoneal administration of streptozocin (STZ) (150 mg/kg, Sigma). One or two weeks after the injection of STZ, blood glucose levels were measured and the mice showing hyperglycemia, i.e. 400 to 500 mg/dL, were used for transplantation. Cells were transplanted into the kidney capsule of anesthetized recipient mice. Alternatively, hyperglycemic Akita mice were used for transplantation. At the indicated time points after transplantation, blood was drawn from the tail vein and the blood glucose level was determined with a glucose meter according to the manufacturer's instructions (Roche Diagnostic). Three months after transplantation, the kidney was subjected immunostaining. Transplantations were performed between 8–12 weeks of age in both model mice.

### Glucose challenge

To exclude insulin added in the culture medium, cultured cells were washed three times with PBS, and subsequently washed with Krebs-Ringer buffer. Krebs-Ringer buffer with a low glucose concentration (50 mg/dL) was added and the cells cultured for 30 min. This low glucose buffer was then collected, and the cells washed with Krebs-Ringer buffer. Krebs-Ringer buffer with a high glucose concentration (500 mg/dL) was added and the cells cultured for 30 min. This high glucose buffer was also collected. The collected buffers were used to measure insulin concentrations by ELISA (Shibayagi) according to the manufacturer's instructions.

### Statistical analyses

Data presented are means ± SEM from at least three independent experiments. Statistical comparisons between two experimental groups were made using Student's paired *t*-test and *P* values<0.05 were considered significant.

## Results

### Development of mouse pancreas and cultivation of fetal pancreatic cells

**Figure 1:**
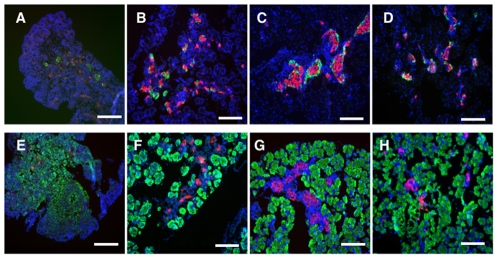
Formation of pancreatic islets and acini during development. Immunohistochemical staining of frozen sections of pancreatic tissues at E14.5 (**A, E**), E16.5 (**B, F**), E18.5 (**C, G**), and neonate (**D, H**). (**A–D**) Glucagon (green) and insulin (red), (**E–H**) amylase (green) and insulin (red) staining. Nuclei were stained with dapi. Bar = 100 µm.

We first investigated when islets are formed by immunostaining the pancreas during mouse development. Several cells in the pancreas at E14.5 expressed amylase, glucagon or insulin, but they were scattered without forming a specific structure (**Fig. 1**). Although insulin- and glucagon-producing cells at E16.5 were in close contact, the typical islet structure was not yet formed. Structures similar to islets in the adult pancreas were clearly observed at E18.5. These results indicate that islets form between E16.5 and E18.5.

**Figure 2:**
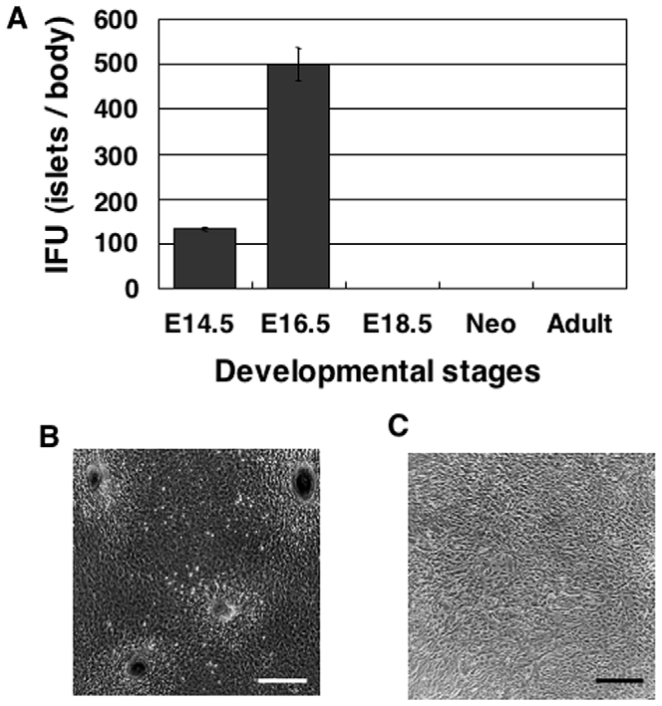
Formation of islet-like spherical cell aggregates *in vitro*. Pancreatic tissues were dissociated and cultured for 14 days. (**A**) Numbers of islet-like spherical structures larger than about 20 µm in diameter formed from one animal. (**B, C**) Photomicrographs of the cultured cells formed from pancreatic tissues at E16.5 (**B**) and E18.5 (**C**). Bar = 300 µm.

To test whether islets can be formed *in vitro*, we cultured fetal pancreatic cells at E16.5, just before islets develop *in vivo*, using a previously reported culture medium for pancreatic cells [6]. We found that E16.5 fetal pancreatic cells proliferated as a monolayer and subsequently formed spherical cell aggregates (SCAs) of heterogeneous size by day 12. We optimized the culture conditions by changing growth factors, extracellular matrics (ECM), source of the cells and so forth, and found that SCA formation was significantly increased by addition of EGF (**Fig. S1**) but not different between collagen and gelatin (data not shown). The fetal pancreatic cell culture system (FPCS) is described in the **Materials and Methods**. Notably, SCAs were most efficiently formed from E16.5 pancreatic cells and less efficiently from E14.5 cells. By contrast, neither E18.5, neonate, nor adult pancreatic cells formed the islet-like structure in FPCS (**Fig. 2**). These results suggest that the islet-forming potential *in vitro* is present only transiently in the fetal pancreas around E16.5, coinciding with our immunostaining results *in vivo*, i.e. that islets are formed between E16.5 and E18.5 (**Fig. 1**). These results are consistent with previous reports in human pancreas [9], [13], [14].

### Characterization of cell aggregates formed *in vitro*

**Figure 3:**
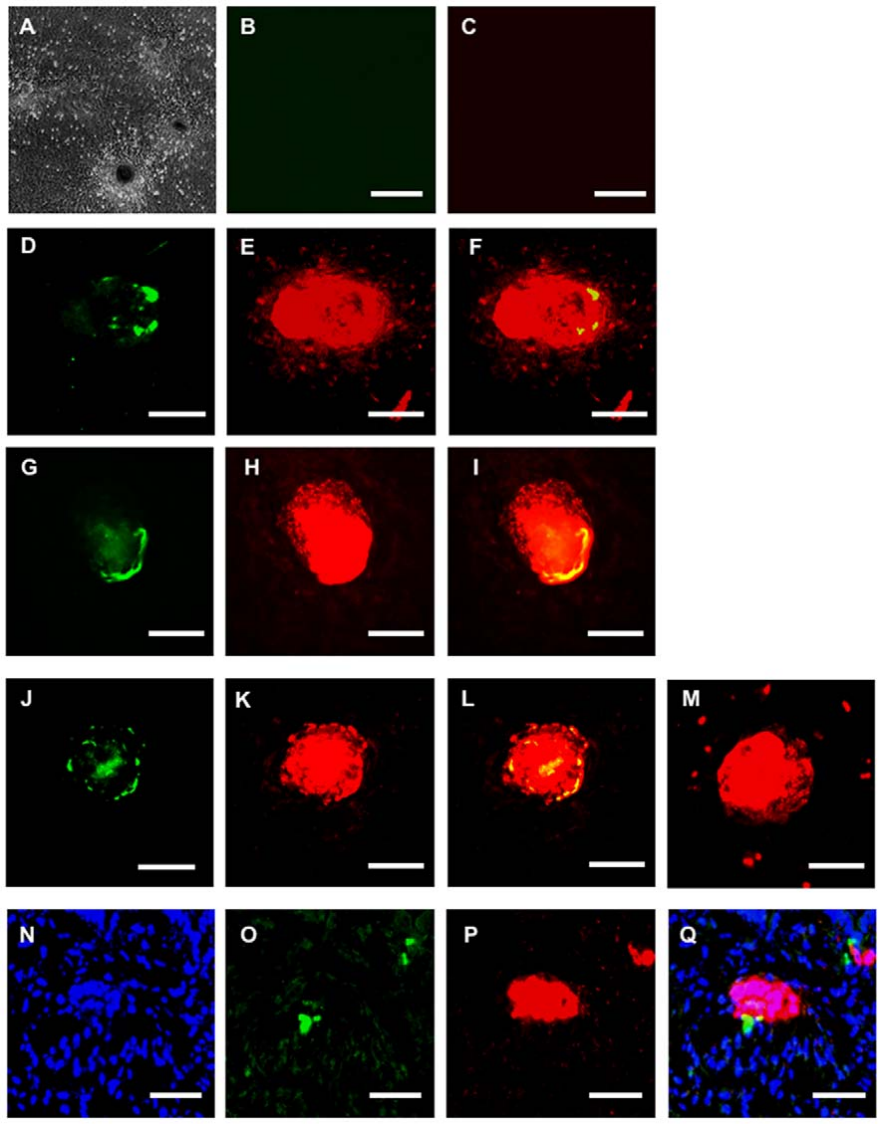
Expression of pancreatic hormones in spherical cell aggregates. (**A**) Photomicrograph of islet-like SCAs. (**B**, **C**) Negative controls of the staining for glucagon, somatostatin, and pancreatic polypeptide (**B**, green), and for insulin (**C**, red). (**D–M**) Staining of glucagon, somatostatin, pancreatic polypeptide, and insulin; glucagon (**D**, green), somatostatin (**G**, green), pancreatic polypeptide (**J**, green), insulin (**E**, **H**, **K**, red), and C-peptide (**M, red**). **F**, **I**, **L** are the merged image of **D** and **E**, **G** and **H**, **J** and **K**, respectively. (**N–Q**) Staining of a frozen section. Dapi (**N**, blue), glucagon (**O**, green), insulin (**P**, red), and a merged view for **N**–**P** (**Q**). Bars = 100 µm.

To investigate whether SCAs formed in FPCS were indeed pancreatic islets, we immunostained the cultured cells. Large SCAs were positively stained for insulin, glucagon, somatostatin, pancreatic polypeptide and also C-peptide (**Fig. 3**). Importantly, insulin-producing cells were surrounded by glucagon-, somatostatin-, or pancreatic polypeptide-producing cells, showing the typical “mantle-core” structure of adult murine pancreatic islets. In contrast, a majority of small SCAs, smaller than 20 µm, produced insulin but not glucagon, similar to islets in adult pancreas. In addition, the mRNA expression of *ins1* (data not shown) and C-peptide (**Fig. 3 M**) in cultured fetal pancreatic cells indicated that the insulin we detected by immunostaining was in fact produced from the cells in SCAs, and not by the absorption of insulin in the culture medium [21]–[25]. By contrast, no acinus-like structure was found in E16.5 cultures, though there were some amylase-producing cells (**Fig. S2**).

### Glycemic control by cultured pancreatic cells

**Figure 4:**
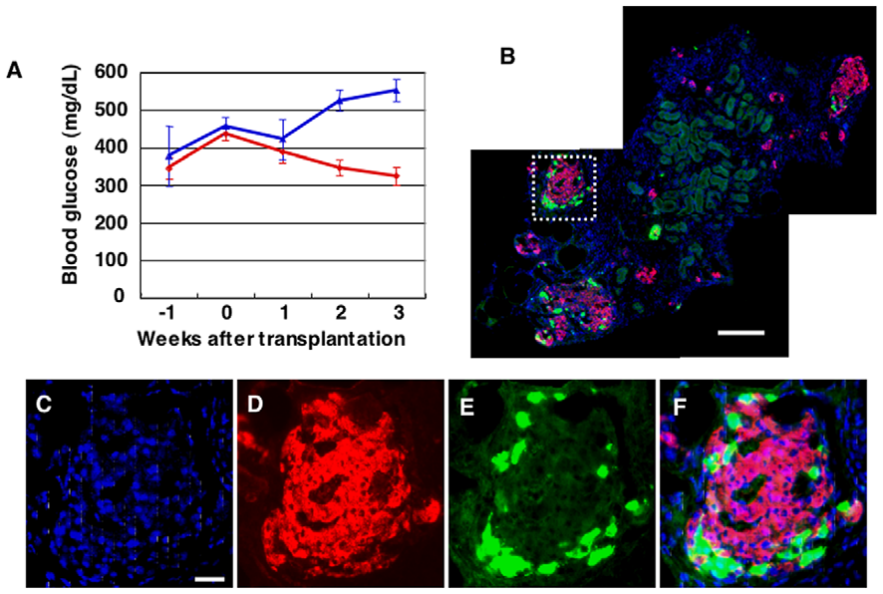
Transplantation of islets formed *in vitro*. (**A**) Blood glucose levels of the hyperglycemic mice transplanted with E16.5 fetal pancreatic cells (blue) or cultured cells (red). Immunostaining of frozen sections of the kidney 3 months after the transplantation; (**B**) Glucagon (green), insulin (red), and dapi (blue). Bar = 100 µm (**C–F**) High magnification of (**B**). Bar = 20 µm.

To evaluate the *in vivo* function of the islets formed *in vitro*, we transplanted them into the kidney capsule of mice with streptozocin (STZ)-induced diabetes. Blood glucose levels improved soon after the transplantation of cultured pancreatic cells, whereas the transplantation of fetal pancreatic cells without cultivation in FPCS failed to reduce the glucose levels (**Fig. 4 A**). Thus, the formation of islets in FPCS is necessary for fetal pancreatic cells to function *in vivo*.

To examine the fate of transplanted islets, we immunostained frozen sections of the recipient kidneys three months after the transplantation (**Fig. 4 B–F**). There were many SCAs in which insulin-producing cells were surrounded by glucagon-producing cells, indicating that islets were maintained in the kidney capsule for at least three months. Notably, no tumor-like structures were found in our experiments. Based on these structural and functional characteristics, we conclude that functional and transplantable islets can be produced *in vitro* from fetal pancreatic cells obtained just before islets form *in vivo*.

### Generation of islets from mouse iPS cells

**Figure 5:**
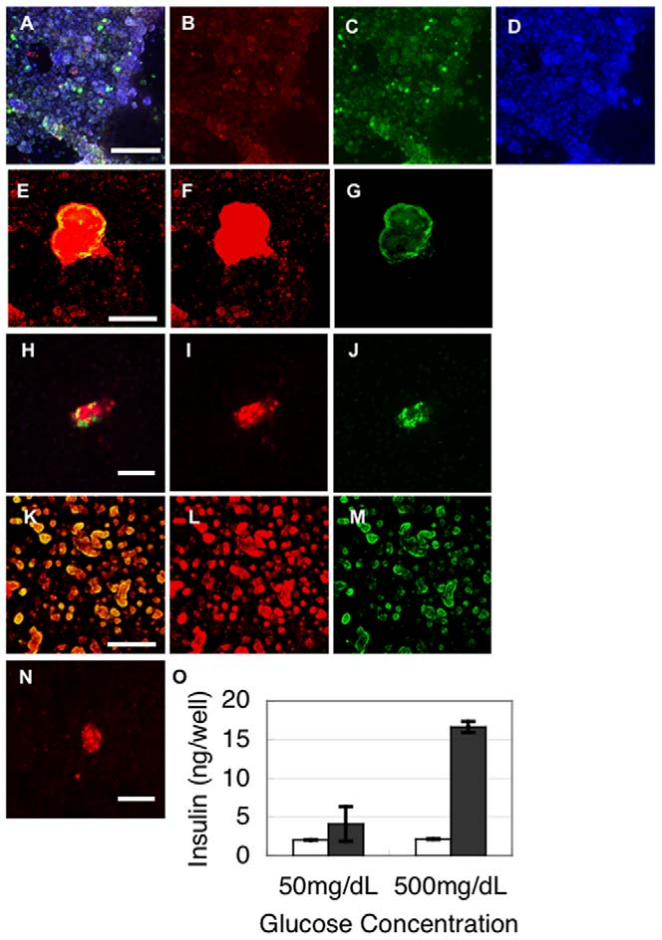
Formation of islets from miPS cells. (**A**–**D**) Immunostaining of pancreatic cells from miPS cells just before cultivation in FPCS. Insulin (**B**, red), glucagon, somatostatin, and pancreatic polypeptide (**C**, green), dapi (**D**, blue) and a merged image (**A**). (**E**–**N**) Immunostaining of large (**E**–**G**), medium (**H**–**J**, **N**), and small (**K**–**M**) islets formed from miPS cells. Glucagon, somatostatin, and pancreatic polypeptide (**G**, **J**, **M**, green), insulin (**F**, **I**, **L**, red), C-peptide (**N**, red). Merged images of **F**, **G** and **I**, **J** and **L**, **M** are shown as (**E**, **H**, **K**) respectively. Bar = 100 µm (**O**) Secretion of insulin before (white) and after (gray) cultivation in the presence of high (500 mg/dL) and low (50 mg/dL) concentrations of glucose. Data presented are means ± SEM (N = 3). Similar results were obtained in three independent experiments.

Previous studies showed that ES cells could be converted *in vitro* to pancreatic hormone-expressing cells [8]–[10]. Those cells formed islet-like clusters a few months after their transplantation into mice [9]. However, islets have never been produced *in vitro* from ES/iPS cells. We therefore considered that the application of FPCS to pancreatic immature cells derived from ES or iPS cells might allow the generation of islets *in vitro* (**Fig. S4**). To test this possibility, we first produced pancreatic cells from either mouse ES or iPS cells according to the modified protocol of previously reported [18]–[20]. We confirmed that although there were some insulin- and/or glucagon- expressing cells scattered in the culture at this point, SCAs were not formed (**Fig. 5 A–D**).

Thereafter, we transferred the cultured cells into FPCS to further induce differentiation and the formation of islets. SCAs were formed at 14 days after replating in FPCS, similar to fetal pancreatic cells. We optimized the culture conditions and found that the formation of islets was dependent on the timing of the replating and plating cell density (**Fig. S3**). Typically, 6567±401 large SCAs and numerous small cell clusters were formed in one well of a 6-well-plate. Immunostaining revealed that the large SCAs expressed insulin and glucagon, showing the islet-like 3D mantle-core structure (**Fig. 5 E–J, N**). The small cell clusters expressed only insulin, similar to those formed from fetal pancreatic cells (**Fig. 5K–M**). Basically the same results were obtained using mouse ES or iPS cells.

### Function of islets derived from mouse iPS cells

To evaluate the function of these islets *in vitro*, iPS-derived cells were challenged with glucose before or after cultivation in FPCS (**Fig. 5 O**). Although cells in both conditions secreted insulin at a very low level at the low glucose concentration, the cells cultured in FPCS with the high glucose concentration secreted about 8 times more insulin. These results indicate that cultivation in FPCS confers on the iPS-derived pancreatic cells the ability to secrete insulin in response to the glucose concentration.

**Figure 6:**
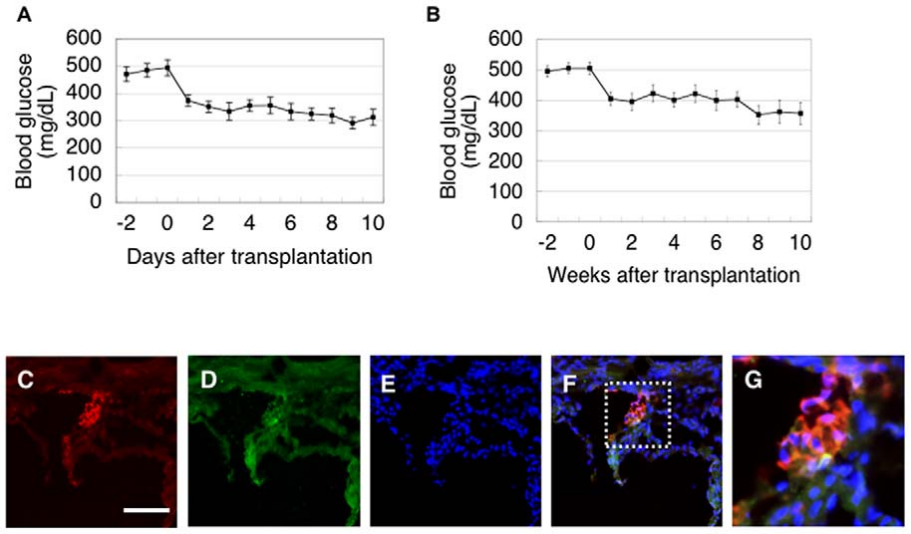
Transplantation of islets formed from miPS cells. (**A, B**) Blood glucose levels of the Akita mice transplanted with islets formed from miPS cells (**A**; glucose levels were measured for 10 days n = 7, **B**; glucose levels were measuered for 10 weeks n = 9). (**C–G**) Immunostaining of frozen sections of kidney three months after transplantation; (**C–F**) insulin (**C, F**, red) and glucagon, somatostatin, pancreatic polypeptide (**D, F**, green) and dapi (**E, F**, blue). (**G**) Higher magnification of (**F**). Bar = 100 µm.

Furthermore, the transplantation of iPS-derived cells in the kidney capsules of hyperglycemic Akita mice quickly reduced blood glucose levels and maintained the low glucose levels at least 10 weeks (**Fig. 6 A, B**). Typically at 9 days after transplantation, the blood glucose levels were reduced to about 300 mg/dL which is close to those in WT littermate mice, i.e. 257.9±11.4 mg/dL under nonfasting conditions (n = 10) (**Fig. 6 A, B**). Histochemical analysis revealed islet-like structures to be present in the kidney capsules three months after transplantation (**Fig. 6 C–G**), indicating that iPS-derived islets can be maintained long term. In addition, no tumor was found in the transplanted mice. Taken together, our culture system allows the generation of functional islets with a unique 3D structure from both fetal pancreatic cells and miPS cells (**Fig. S4**).

## Discussion

We show in this paper that islets can be formed *in vitro* from fetal pancreatic cells and iPS cells. The islets exhibit a mantle-core structure, i.e. insulin-producing cells surrounded by glucagon-producing cells, a distinctive feature of murine pancreatic islets. Islets formed *in vitro* secrete insulin in response to the glucose concentration, and improve hyperglycemia when transplanted in the kidney capsules of hyperglycemic mice. Although there are several reports describing the differentiation of pancreatic hormone-producing cells from ES cells as well as other cell sources [6]–[12], to our knowledge this is the first report showing the generation of functional islets with the proper 3D structure *in vitro* from iPS cells. Since transplantation requires this 3D structure, the generation of islets *in vitro* from iPS cells paves the way to providing a sufficient quantity of islets for clinical application.

Previous studies showed that there are common progenitors for endocrine and exocrine cells in E11.5 pancreatic tissues [4], and that islet-like structures developed in organ cultures of E11.5 pancreas [26]–[28]. In our culture, islets were formed most efficiently from E16.5 pancreatic tissues and much less efficiently from E14.5 pancreas, however no islet-like structure was formed from E12.5 and E18.5 pancreatic tissues. These results suggest that the endocrine progenitors rather than common progenitors are responsible for the islet-forming potential in FPCS.

The number of progenitors in the fetal pancreas determines the size of the adult pancreas [4]. It has been estimated that there are about 2,500 islets including insulin-producing small cell clusters in the adult pancreas of a C57BL/6 mouse [29], [30]. In FPCS, 499±36.3 islets together with many small cell clusters were formed from a single embryo at E16.5, indicating that the efficiency is reasonably high. However, it still remains unclear whether the number of islets formed in FPCS directly correlates with the number of islet progenitors. As fetal pancreatic tissues were dispersed before the culture and islets were formed *in vitro* in a cell density-dependent manner, islets may be produced by the assembly of immature endocrine cells [31] rather than from a single progenitor cell. While the precise nature of the islet progenitor remains unknown, the culture system will provide a means to characterize the progenitors.

An interesting and important finding in this paper is that there is a critical window of time that allows the formation of 3D islet structures *in vitro*. By adopting FPCS to pancreatic cells at the appropriate stage of differentiation from iPS/ES cells, functional islets were developed *in vitro*. Previous reports showed that by the sequential addition of Activin/Wnt3, FGF, and FGF/Noggin/cyclopamin, human ES cells were converted to insulin-producing cells without showing any islet-like structures [8], likely corresponding to the pancreatic cells at 6–9 weeks of age [32], [33]. The human fetal pancreas at this age is probably equivalent to an E14.5–16.5 mouse pancreas, which contains only a few hormone-expressing cells that do not respond to glucose [34]. Although pancreatic cells derived from ES cells did not form any islet-like structures *in vitro*, functional islets were formed in kidney capsules of immune-deficient mice several months after the transplantation [9]. Taken together, it is suggested that there is a critical window of time for pancreatic cells, just before islet-genesis, to form islets *in vitro*. Our results using mouse iPS cells are consistent with these studies, however, they are significantly different from previous reports because islet-like structures are formed in our culture system and those cells produce insulin in response to glucose. Furthermore, the transplantation of those cells immediately improved the blood glucose level in hyperglycermic mice. In conclusion, by applying FPCS to iPS-derived immature pancreatic cells, transplantable islets can be generated *in vitro*.

## Supporting Information

Figure S1 **Effect of growth factor in islet formation** ***in vitro*** **.** Effect of growth factors for islet formation. EGF is the only effective factor for islet formation within all growth factors and cytokines we have. However, insulin and GLP-1 has a potential to stabilize the culture, we use the combination of EGF, insulin, and GLP-1. * significantly different. (p<0.05).
(TIF)

Figure S2 **Acini were not formed in this culture system.** (**A**–**C**) Amylase staining (**B**, green), Dapi staining (**A**, blue), and merged image (**C**) of (**A** and **B**) of the multiple layers formed from E16.5 pancreatic cells *in vitro*. Scale bars, 200 µm.
(TIF)

Figure S3 **Timing and seeding density is important for islet formation** ***in vitro*** **.** **A**, Formation of islets at different replating timing. The islet forming efficiency was higher at later replating time points, however, we could not replate after 8 days in the same condition (n = 4). **B**, Islet forming frequency depends on seeding cell density. There is a very narrow window of islet formation for forming islets at high efficiently.
(TIF)

Figure S4 **Diagram of the culture system for producing islets from iPS cells.** Islets are formed *in vitro* from pancreatic tissues just before the islet formation *in vivo*. iPS cells are induced to differentiate to pancreatic cells sequentially by activin, retinoic acid, bFGF, bFGF+nicotinamide and those pancreatic cells are then replated in culture medium that induces islet formation from fetal pancreatic cells.
(TIF)
