# Supplementary figures and images for: Generation of Glucose-Responsive Functional Islets with a Three-Dimensional Structure from Mouse Fetal Pancreatic Cells and iPS Cells In Vitro
Source: PLoS One. 2011 Dec 1;6(12):e28209. doi: 10.1371/journal.pone.0028209 (PMC3228734; doi:10.1371/journal.pone.0028209)

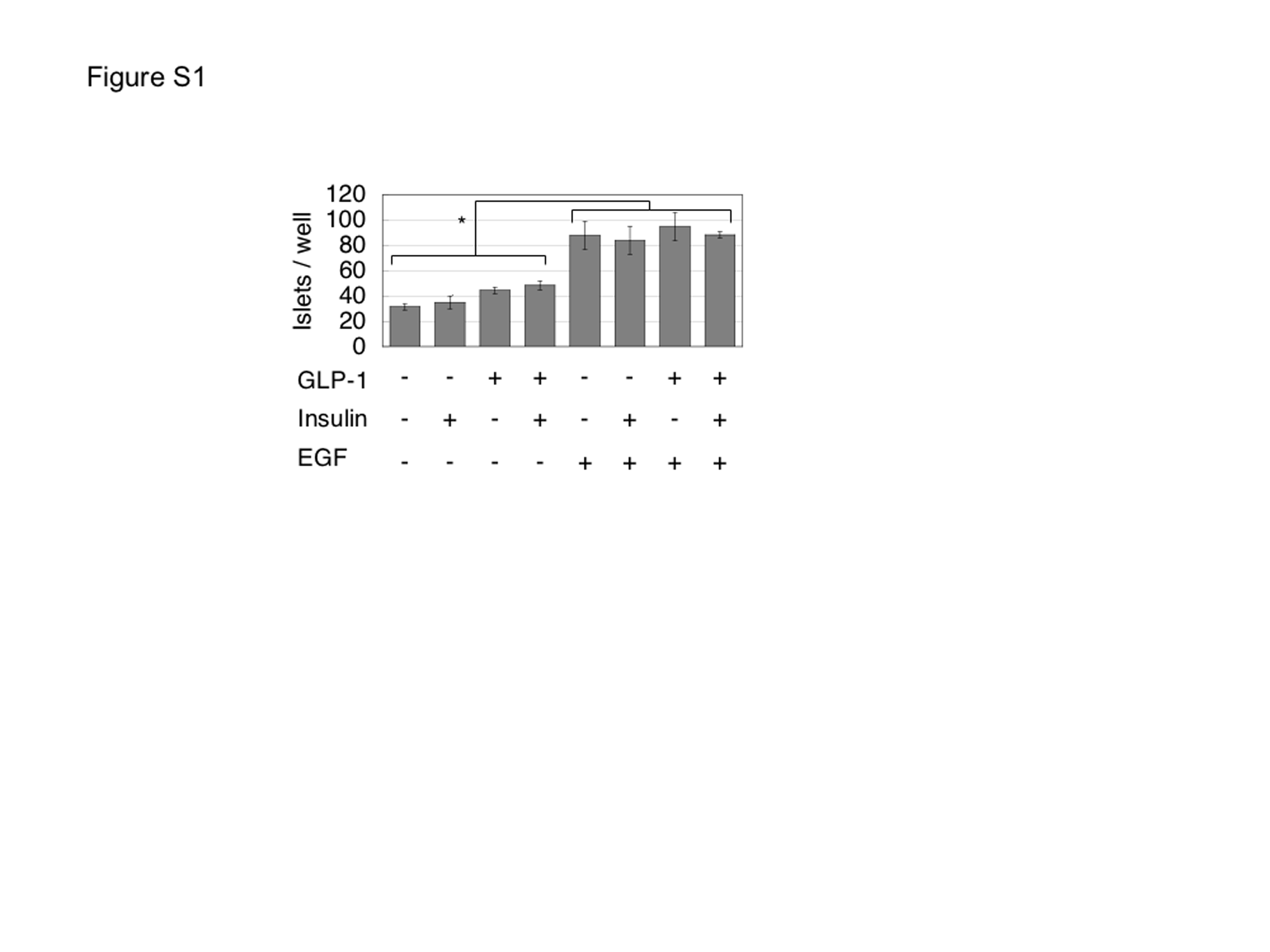

Supplement: Figure S1 — Effect of growth factor in islet formation in vitro . Effect of growth factors for islet formation. EGF is the only effective factor for islet formation within all growth factors and cytokines we have. However, insulin and GLP-1 has a potential to stabilize the culture, we use the combination of EGF, insulin, and GLP-1. * significantly different. (p<0.05). (TIF) [file pone.0028209.s001.tif]

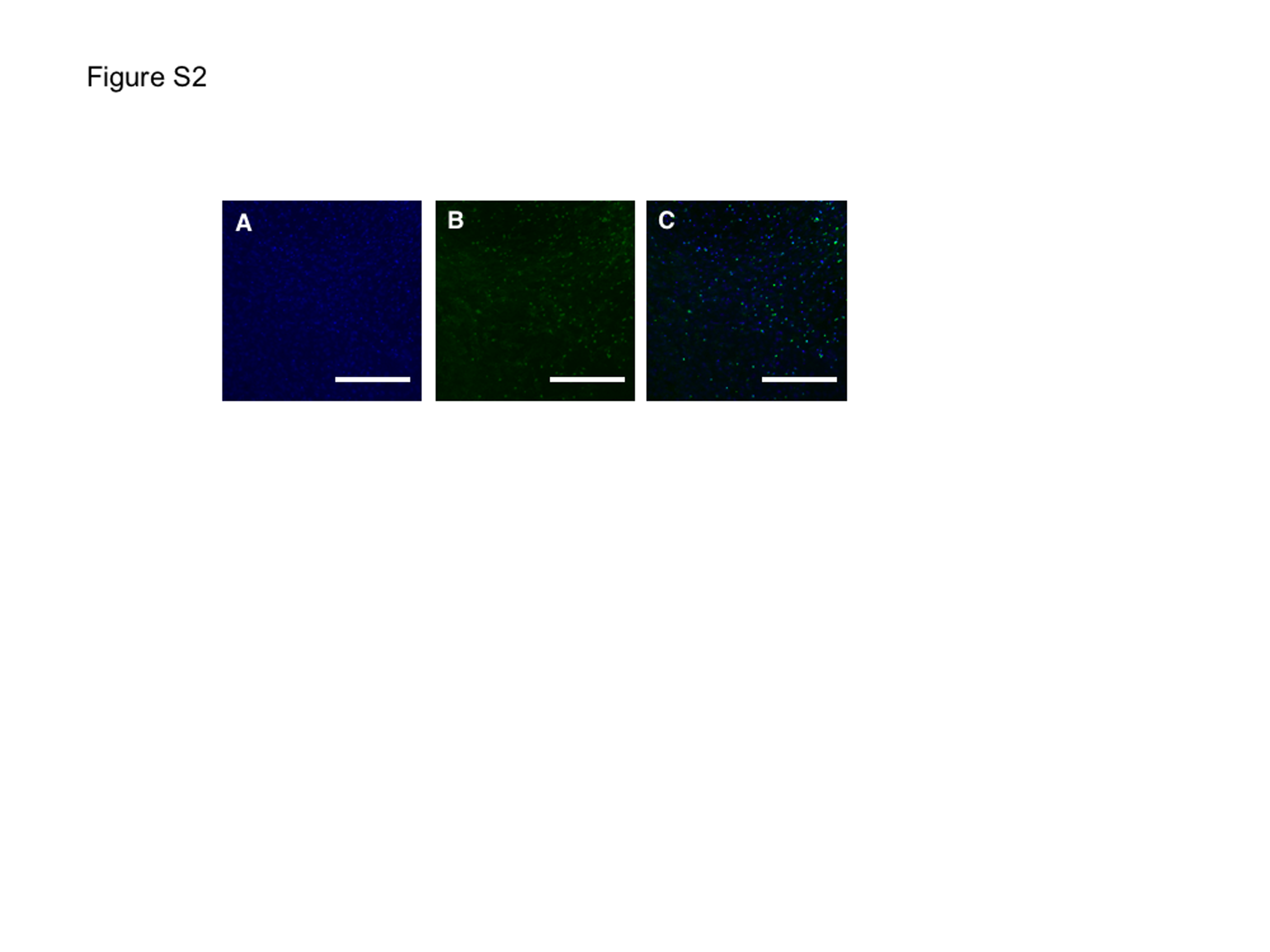

Supplement: Figure S2 — Acini were not formed in this culture system. (A–C) Amylase staining (B, green), Dapi staining (A, blue), and merged image (C) of (A and B) of the multiple layers formed from E16.5 pancreatic cells in vitro. Scale bars, 200 µm. (TIF) [file pone.0028209.s002.tif]

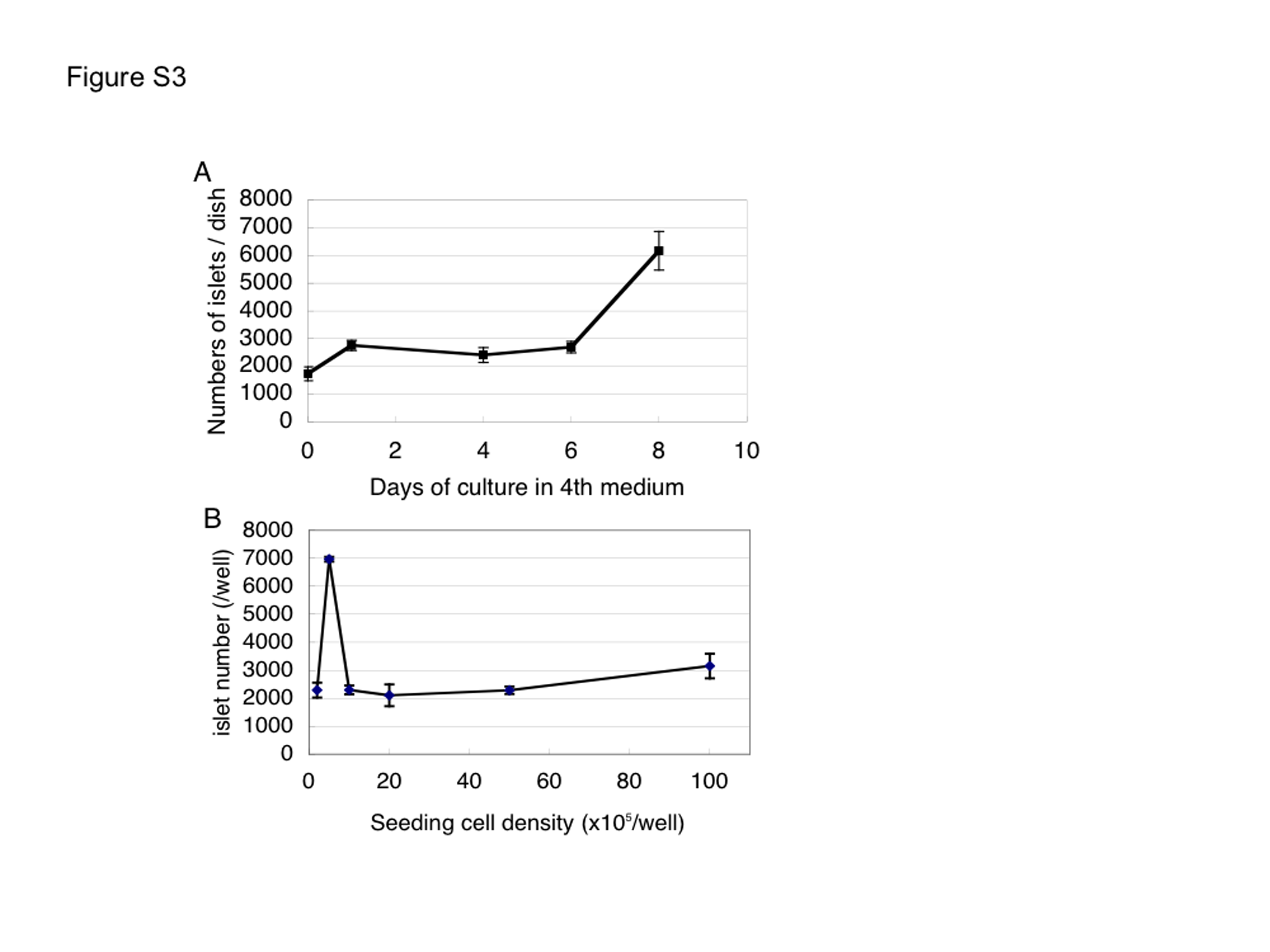

Supplement: Figure S3 — Timing and seeding density is important for islet formation in vitro . A, Formation of islets at different replating timing. The islet forming efficiency was higher at later replating time points, however, we could not replate after 8 days in the same condition (n = 4). B, Islet forming frequency depends on seeding cell density. There is a very narrow window of islet formation for forming islets at high efficiently. (TIF) [file pone.0028209.s003.tif]

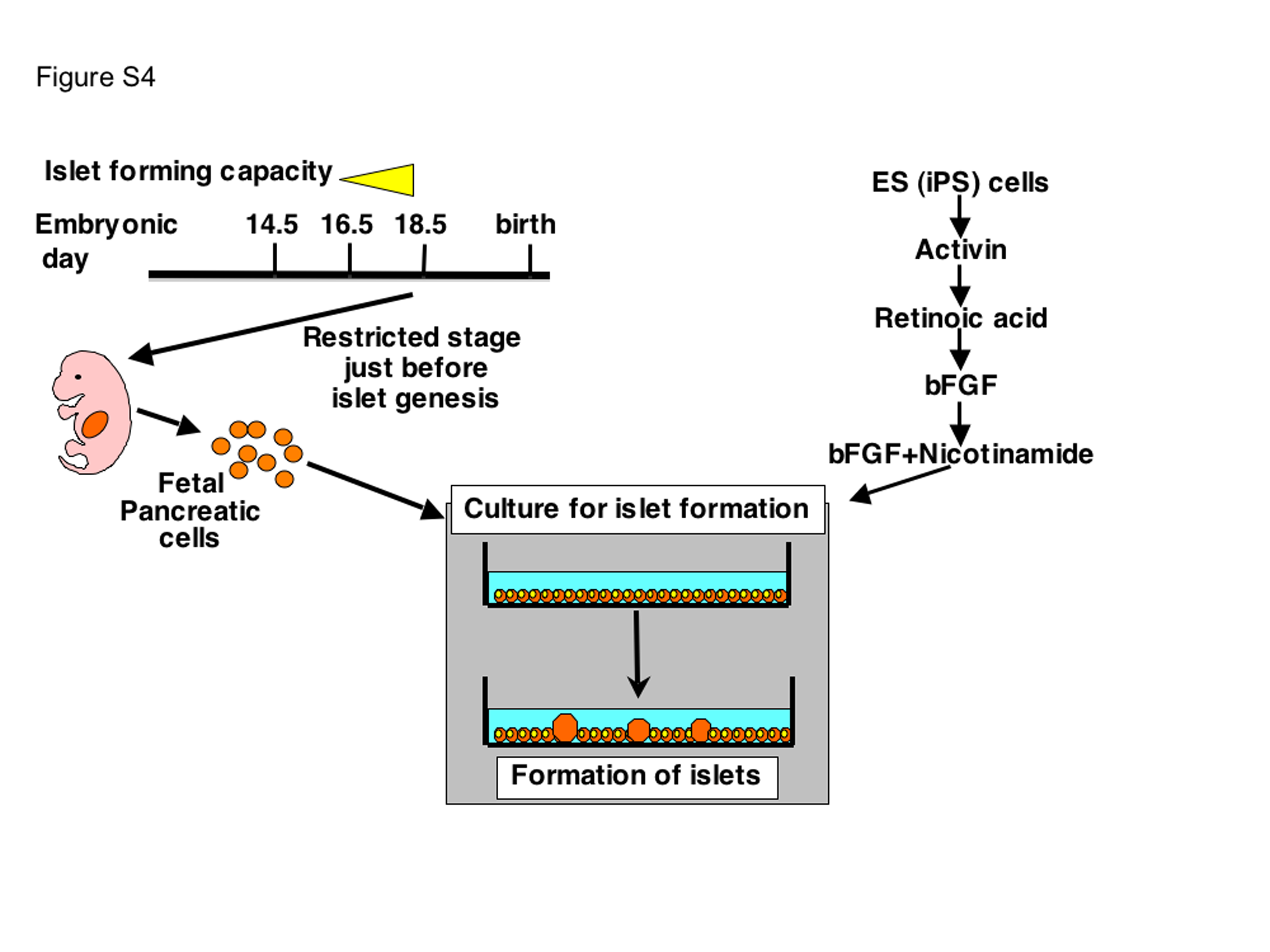

Supplement: Figure S4 — Diagram of the culture system for producing islets from iPS cells. Islets are formed in vitro from pancreatic tissues just before the islet formation in vivo. iPS cells are induced to differentiate to pancreatic cells sequentially by activin, retinoic acid, bFGF, bFGF+nicotinamide and those pancreatic cells are then replated in culture medium that induces islet formation from fetal pancreatic cells. (TIF) [file pone.0028209.s004.tif]
